# Anisotropic metamaterials for scalable photonic integrated circuits: a review on subwavelength gratings for high-density integration

**DOI:** 10.1515/nanoph-2024-0627

**Published:** 2025-03-31

**Authors:** Yosep Shin, Kyungtae Kim, Jaewhan Lee, Saman Jahani, Zubin Jacob, Sangsik Kim

**Affiliations:** School of Electrical Engineering, Korea Advanced Institute of Science and Technology, Daejeon 34141, Republic of Korea; Graduate School of Quantum Science and Technology, Korea Advanced Institute of Science and Technology, Daejeon 34141, Republic of Korea; ASML, Wilton, CT 06897, USA; School of Electrical and Computer Engineering, Purdue University, West Lafayette, IN 47907, USA; School of Electrical Engineering and Graduate School of Quantum Science and Technology, Korea Advanced Institute of Science and Technology, Daejeon 34141, Republic of Korea

**Keywords:** anisotropic metamaterials, subwavelength gratings (SWGs), photonic integrated circuits (PICs), silicon photonics, integration density

## Abstract

Photonic integrated circuits (PICs) are transforming optical technology by miniaturizing complex photonic elements and systems onto single chips. However, scaling PICs to higher densities is constrained by optical crosstalk and device separation requirements, limiting both performance and size. Recent advancements in anisotropic metamaterials, particularly subwavelength gratings (SWGs), address these challenges by providing unprecedented control over evanescent fields and anisotropic perturbations in PICs. Here we review the role of anisotropic SWG metamaterials in enhancing integration density, detailing two foundational mechanisms – skin depth engineering and anisotropic perturbation – that mitigate crosstalk and enable advanced modal controls. We summarize their applications within four key functions: confinement manipulation, hetero-anisotropy and zero-birefringence, adiabatic mode conversion, and group velocity and dispersion control, showing how each benefits from distinct SWG properties. Finally, we discuss current limitations and future directions to expand the full potentials of anisotropic SWG metamaterials, toward highly dense and scalable PICs.

## 1 Introduction

Over the last decade, photonic integrated circuits (PICs) have advanced significantly, offering unprecedented research and industrial opportunities [1], [2], [3], [4], [5]. As PIC technology continues to progress, the research scope has expanded from fundamental science toward systematic implementations, including photonic AI accelerators [6], [7], [8], [9], programmable PICs [10], [11], [12], [13], and emerging industrial applications such as optical interconnects [14], [15], [16], co-packaged optics [17], [18], and LiDAR [19], [20], [21]. As PIC applications become increasingly complex, the need for higher chip density with more components has become critical. However, achieving high-density integration faces significant challenges, particularly in managing optical crosstalk. Crosstalk, arising from evanescent field overlap between adjacent waveguides or devices, introduces noise that degrades the signal-to-noise ratio (SNR) across the system. While optical crosstalk may be manageable in small-scale systems, it becomes a serious limitation when scaled to hundreds or thousands of components. A tradeoff exists between reducing crosstalk with a large device separation and pursuing compact integration, imposing scalability constraints on PICs.

Meanwhile, the field of metamaterials has explored new ways to overcome such scaling limitations. All-dielectric metamaterials, which alternate dielectric layers to exhibit unique anisotropic properties along different axes, have demonstrated promising applications in integrated photonics. Effective medium theory (EMT), which models these metamaterials as homogeneous media, has been pivotal in predicting and designing anisotropic behaviors within these structures. Building on this framework, all-dielectric anisotropic metamaterials have emerged as a promising approach to manipulate skin depth in integrated waveguides, enabling crosstalk suppression and even achieving complete crosstalk elimination through anisotropic perturbation effects. Anisotropic metamaterials can be formed using transition metal dichalcogenides (TMDCs) [22], [23] and subwavelength gratings (SWGs). In PICs, anisotropic metamaterials are often realized by SWGs that are straightforward to fabricate and highly compatible with monolithic PIC platforms [24], [25], [26], [27], [28], [29]. This SWG-based approach has supported significant progress in high-density PIC integration, as researchers develop novel devices that benefit from both anisotropy and tailored confinement properties [28], [29], [30], [31], [32], [33], [34], [35].

In this context, this review article explores the foundations and applications of anisotropic SWG metamaterials, with a particular focus on their role in enhancing integration density for high-performance PICs. Since there are already a couple of excellent review papers on SWG fundamentals covering a broad range of applications [27], [28], [29], [30], [31], this review instead focuses on the role of anisotropic metamaterials in increasing integration density while highlighting recent advances in SWG innovations. In Section 2, we provide an overview of various EMT models and how they relate to the anisotropic behaviors achievable with SWG metamaterials. This section also introduces the range of modal engineering possibilities enabled by different SWG geometries. Section 3 discusses the principles and implementations of skin depth engineering using anisotropic metamaterials across different coordinate systems. Section 4 then delves into anisotropic perturbation techniques for achieving zero crosstalk, detailing the physics underlying these methods. Section 5 summarizes recent advancements in using anisotropic SWGs in PIC components, categorized by three core engineering approaches – index, bandgap, and anisotropy engineering. Each approach is also classified by its function in confinement manipulation, hetero-anisotropy and zero-birefringence, adiabatic mode conversion, and group velocity and dispersion control. In Section 6, we conclude with an outlook on the future potentials with anisotropic SWGs in PICs, highlighting avenues for further exploration in scaling and device performance.

## 2 Effective medium theory for anisotropic metamaterials

Periodically structured media are well-known for exhibiting unique electromagnetic properties, including anisotropy [36], [37], [38], [39], [40], [41], [42]. SWGs have emerged as a powerful tool for engineering anisotropy in optical metamaterials, where their subwavelength-scale periodicity induces strong birefringence [27]. This strong birefringence, which is challenging to achieve with natural materials, enables SWGs to support various applications, such as polarization controllers. However, designing these anisotropic electromagnetic characteristics directly through structural manipulation with Maxwell’s equations is computationally intensive. Here, EMT offers a solution by approximating such structured media as homogeneous, anisotropic materials. In the deep-subwavelength regime, where the wavelength of light is much larger than the structure’s periodicity, first-order EMT approximations are often employed, providing reliable estimates of the anisotropic indices. Since our focus is on PIC composed of all-dielectric materials operating at optical frequencies, the permeability is assumed to be isotropic and equal to vacuum permeability. It is worth noting that modal birefringence is ubiquitous in all waveguide structures without rotational symmetry, and modifying waveguide geometry inherently alters modal birefringence [43], [44]. However, this conventional variation in modal birefringence is constrained by standard waveguiding mechanisms and is not the focus of our study.

**Figure 1:**
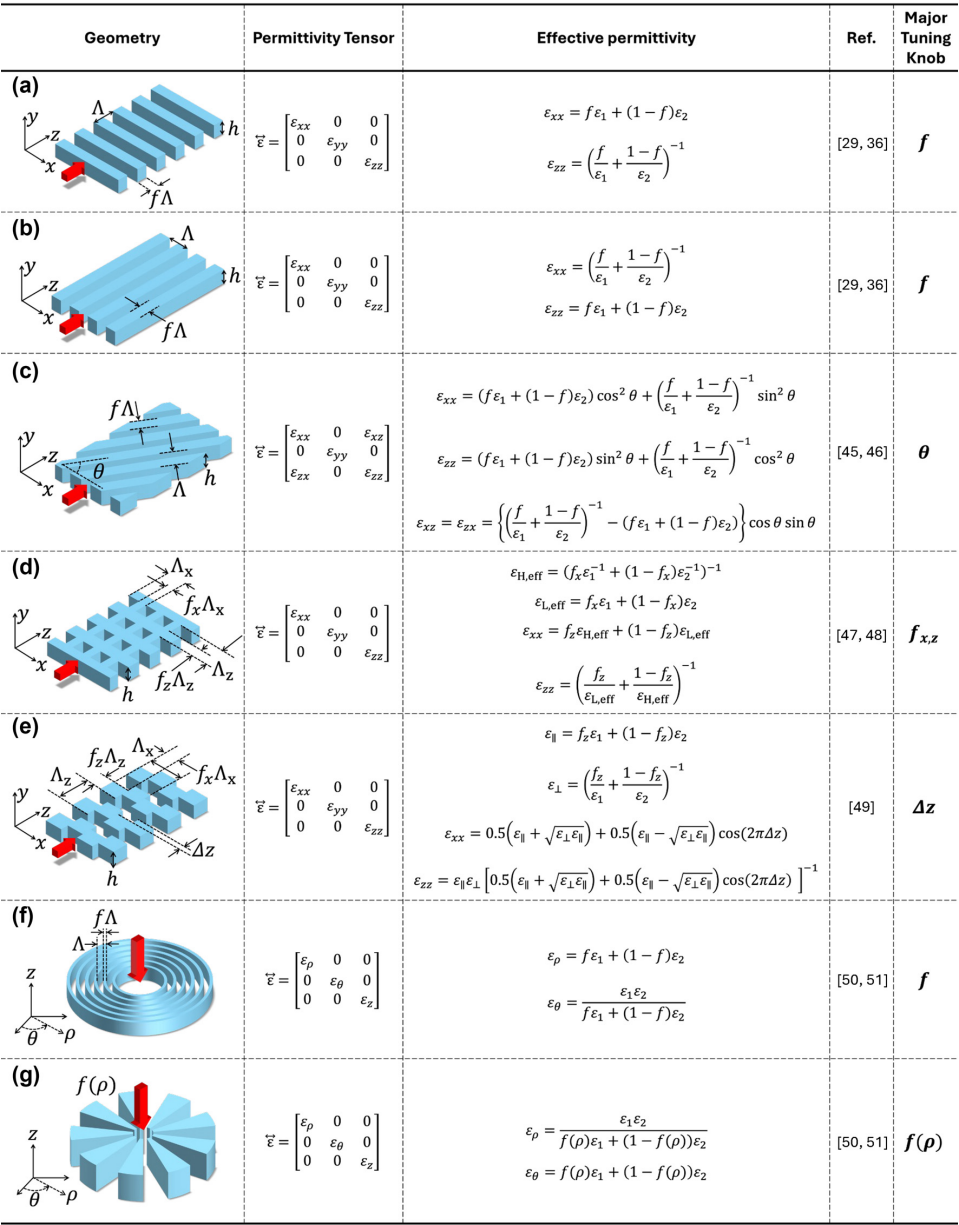
Summary of effective medium theories (EMTs) for modeling anisotropic SWG metamaterials. Different SWG geometries lead to distinct anisotropic dielectric responses, which can be modeled using EMTs. Each structure illustrates how periodicity along different axes influences the dielectric tensor and overall anisotropic properties. (a) A uniaxial SWG structure with periodicity along the *z*-axis, and (b) periodicity along the *x*-axis, each yielding corresponding anisotropic responses [29], [36]. (c) A generalized 1D periodic SWG representation, incorporating the tilt angle *θ* [45], [46]. (d) Biaxial SWGs with periodicities along both the *x*- and *z*-axes, where effective permittivity is calculated in two steps: first along the *z*-axis, then along the *x*-axis [47], [48]. (e) Bricked SWGs with a grating offset Δ*z*, where displacement adjustments modify the permittivity tensor, allowing for larger feature sizes and enhanced fabrication feasibility [49]. Here, the duty cycles, *f*_*x*_ and *f*_*z*_ along *x*- and *z*-axes are assumed to be 0.5. (f, g) Circular SWGs, with EMTs represented in cylindrical coordinates using *ɛ*_*ρ*_ and *ɛ*_*θ*_, where the periodicity is along the (f) *ρ* and (g) *θ* [50], [51]. Note that in all cases, the thickness of the film and cladding must be carefully considered for an accurate determination of *ɛ*_*yy*_. However, it is often assumed to be infinite in the *y*-direction, leading *ɛ*_*yy*_ to be set equal to the parallel component, such as *ɛ*_*yy*_ = *ɛ*_*xx*_ in (a) and *ɛ*_*yy*_ = *ɛ*_*zz*_ in (b).

Figure 1 illustrates various SWG types and their corresponding effective permittivities, modeled as second-rank tensors in the quasi-static limit [36], [45], [46], [47], [48], [49], [50], [51]. Each geometry exhibits distinct anisotropic behavior based on its periodic structure, showing how the dielectric tensor varies with geometric parameters such as filling fraction *f* (or duty cycle), orientation angle *θ*, and offset Δ*z*. The SWG structures are assumed to extend infinitely, with finite film thicknesses and infinite top and bottom layers. These assumptions allow a concise representation of the anisotropic properties, facilitating the use of EMT. It is worth noting that, while periodicity establishes metamaterials represented by EMT, the theory itself depends mainly on the filling fraction and material properties. Periodicity does not directly alter the material properties as described by EMT but instead influences the accuracy of EMT in practical realizations. As the periodicity becomes smaller relative to the wavelength, the structure more closely aligns with the ideal assumptions of EMT, while larger periodicity leads to greater deviations. Instead, periodicity plays a dominant role in determining the bandgap properties of SWGs, which are not explicitly described by EMT.

Figure 1(a) shows periodically arranged slabs with dielectric strips along the *z*-axis, creating an anisotropic permittivity similar to that observed in crystalline structures. While isotropic media have equal diagonal components in their permittivity tensor, anisotropic media exhibit unequal diagonal components. In all-dielectric case, this configuration always exhibits *ɛ*_*xx*_ > *ɛ*_*zz*_, resulting in a lower effective modal index, which corresponds to a lower confinement and a larger modal area. By carefully engineering the filling fractions and SWG distributions, it has been widely applied in fiber-to-chip edge couplers [24], [25], [26], [52], [53], [54] and modal converters [55], [56]. Rotating Figure 1(a) by 90° exchanges *ɛ*_*xx*_ and *ɛ*_*zz*_, as in Figure 1(b). This SWG configuration, where *ɛ*_*xx*_ < *ɛ*_*zz*_, can suppress the skin depth of the TE-guided mode when it is used as a cladding, a topic that will be further discussed in Section 3. It can also be used for anisotropic perturbation to achieve zero-crosstalk, which will be discussed in Section 4.

The configurations in Figure 1(a) and (b) can be generalized by introducing the orientation angle (or tilt angle) *θ*, as in Figure 1(c) [45], [46]. With this tilted SWG configuration, off-diagonal elements appear in the permittivity tensor, which is not manifest in the previous configurations. These off-diagonal elements cause the displacement field $\vec{D}$ to be determined by the superposition of two electric field components, e.g., *D*_*x*_ = *ɛ*_*xx*_ *E*_*x*_ + *ɛ*_*xz*_ *E*_*z*_. The ability to control anisotropic behavior by adjusting the tilt angle *θ* provides an additional degree of freedom for engineering the anisotropy of SWG metamaterials. While precise fabrication resolution is required to control filling fractions, tuning anisotropy via tilt angle offers greater robustness during fabrication. This has been used for advanced polarization handling, e.g., as in polarization-dependent [57] and – independent [58] Bragg gratings.

By combining the configurations in Figure 1(a) and (b), two-dimensional SWGs are also possible, as in Figure 1(d). Here, two different (2D) periodicities and filling fractions are defined for the *x* and *z* axes. The homogenization technique introduced before can be applied consecutively [47]: first, the effective permittivities of the high-index material *ɛ*_1_ and low-index *ɛ*_2_ material can be approximated; for example, $\varepsilon_{H,e\mathrm{ff}}={\left(f_{x}\varepsilon_{1}^{-1}+\left(1-f_{x}\right)\varepsilon_{2}^{-1}\right)}^{-1}$ and *ɛ*_L,eff_ = *f*_*x*_ *ɛ*_1_ + (1 − *f*_*x*_)*ɛ*_2_ for TE mode. Homogenization can then be applied along the *z*-axis to obtain the effective permittivity for the entire structure. This 2D SWG approach expands more independent control of *ɛ*_*xx*_ and *ɛ*_*zz*_, whereas a single parameter adjustment typically affects both. This stepwise homogenization has been applied in designing zero-birefringence grating couplers that operate for both TE and TM modes simultaneously [48].

The 2D-SWG configuration in Figure 1(d) can be further refined as in Figure 1(e) by introducing a slight shift Δ*z* in the grating segments. This configuration, known as bricked-SWG, adopts a Manhattan-like geometry that enhances fabrication feasibility [49]. Unlike 2D SWGs, this design can be realized by pixel dimensions as small as 150 nm × 150 nm, allowing wafer-scale manufacturing while maintaining precise control over anisotropy. The transverse shifting Δ*z* enables the structure to function as a biaxial crystal, allowing effective permittivity tuning without requiring small-feature etching.

SWGs can also be extended to cylindrical coordinates, as illustrated in Figure 1(f). This configuration has been extensively studied for optical hyperbolic metamaterials by alternating dielectric and metallic layers [50], though all-dielectric implementations in PIC platforms remain relatively unexplored. Cylindrical SWGs hold promise for anisotropic control and may offer valuable applications in emitters and radiation control.

The homogenization techniques introduced above rely on the quasi-static limit approximation. First-order EMT provides a straightforward approximation for deeply subwavelength structures but loses accuracy as the periodicity approaches near the wavelength or when dealing with more complex structures. The transition from quasi-static to more dynamic regimes is not well-defined, often leading to inaccuracies when applying first-order EMT near these boundaries. In such cases, higher-order homogenization methods better capture the structural details and periodic effects that first-order EMT overlooks. These methods use a Fourier series expansion of the dielectric function along the periodic structure, expressed as $\varepsilon \left(z\right)=\sum_{p}{\hat{\varepsilon }}_{p}e^{\mathrm{ipGz}}$ , where ${\hat{\varepsilon }}_{p}$ are the Fourier coefficients of the periodic permittivity, and $G=\frac{2\pi }{a}$ is the reciprocal lattice vector [59]. Such Fourier-expanded higher-order approaches provide more accurate estimations of the effective refractive indices for both TE and TM, especially when first-order approximations become inadequate [37], [38]. Studies on the applicability of EMT to 2D SWGs indicate that EMT accurately models structures far below the wavelength but breaks down as feature sizes approach the wavelength [60]. Similar limitations arise in multilayer dielectrics, where nanoscale thickness variations affect light transmission near the critical angle [61]. Advanced methods, such as Rytov transcendental equations, account for resonant behaviors in photonic lattices by predicting multiple effective refractive indices at a single wavelength, enabling precise modeling of broadband reflectors and polarizers [62]. The operator-based effective medium approach further refines EMT by including effects from electric dipoles, chirality, and higher-order interactions, offering improved accuracy for complex layered systems [63].

While higher-order models and refined analytical approaches offer greater accuracies, they may not always meet practical needs, where simplicity and efficiency are often prioritized. As a first step, established first-order EMTs provide essential insight into physical phenomena and allow for identifying key trends in device behavior. However, when precision is required – particularly for complex geometries – comprehensive modeling of the actual structures using numerical tools like FDTD or FEM becomes indispensable. Most research in this field adopts a staged approach: EMT guides preliminary analysis and trend prediction, while detailed numerical simulations refine the design parameters and optimize device performance. This combination of EMT-based analysis with full simulations supports a strategic approach in the field, offering a balanced way that maximizes accuracy and computational efficiency. This hybrid approach enables researchers and engineers to move from theoretical insights to practical device implementations, bridging simplified models and real-world applications.

## 3 Skin-depth engineering for evanescent field control

Wave propagation in anisotropic media was initially studied in 1,669 by Rasmus Bartholin, who discovered birefringence in calcite, which is a natural anisotropic material [64]. Later, in 1801, the French physicist Etienne-Louis Malus further developed the understanding of birefringence by discovering the phenomenon of polarization, which helped to explain the behavior of light in birefringent materials [65]. Birefringence is used in many optical devices, such as liquid-crystal displays, wave plates, modulators, and tunable filters, to name a few.

**Figure 2:**
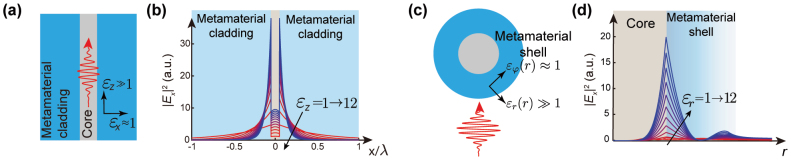
Skin depth engineering with field enhancement. Depending on the configuration, either the suppression or extension of the skin depth can lead to a significant field enhancement. (a) Slab or cylindrical waveguides composed of an isotropic core surrounded by anisotropic metamaterial cladding. Reducing the skin depth results in the field confinement inside the waveguide core. If the anisotropy is strong enough, it is possible to confine light below the diffraction limit [73]. (b) Field intensity of the waveguide mode normalized to the total power. The core is glass (*n* = 1.45) with a size of 0.1*λ* where *λ* is the operating wavelength in vacuum. The transverse component of the permittivity *ɛ*_*x*_ is 1. As we increase the anisotropy of the cladding, light decays faster in the cladding, which leads to field confinement inside the core even though the core size is in the subwavelength regime. (c) Scattering of an incident field by a cylindrical or spherical nanoparticle (nano-antenna) composed of a low-index core and anisotropic metamaterial shell. (d) Scattered field intensity of the 5th TM resonant mode when the structure is excited by a plane wave. The core radius and the shell radius are 0.5 μm and 1.1 μm, respectively. The shell has a graded-index profile such that *ɛ*_*r*_ = 1 at the outer interface. As anisotropy increases, the field intensity at the core/shell interface is enhanced. A comparable effect is observed across other electric (TM) modes [74].

The behavior of non-propagating waves (also known as evanescent fields) in anisotropic media has been explored recently after the invention of metamaterials when many researchers demonstrated strong anisotropy in metal-dielectric structures. Anisotropy in hyperbolic materials (also known as indefinite media [66]) can allow the propagation of high-*k* wavevectors (*k* > *k*_0_), which are evanescent in vacuum [67], [68]. This allows for a large density of states [69] and sub-diffraction lenses [70]. However, the impact of all-dielectric anisotropy on evanescent fields has been studied less. In 2012, we showed that strong anisotropy can also control the skin depth of evanescent fields [71], [72], which can lead to sub-diffraction light confinement in transparent media [73] as illustrated in Figure 2(a) and (b).

### 3.1 Cartesian coordinates

In conventional total internal reflection (TIR), when a propagating light in a dielectric is reflected at the interface of an infinite dielectric with a lower refractive index, light is totally reflected back if the incident angle is larger than a critical angle, which depends on the index ratio of the two mediums: *θ*_*c*_ = arcsin(*n*_2_/*n*_1_), where *n*_1_ and *n*_2_ are the refractive index of the first and the second mediums, respectively. When TIR happens, light evanescently decays in the second medium. The skin depth is the distance at which the field magnitude drops to 1/*e*. Skin depth defines the fundamental limit of light confinement in dielectric waveguides and resonators. The skin depth, *δ*, in isotropic media only depends on the index contrast and the incident angle:

(1) $$\delta =\frac{1}{k_{0}}\frac{1}{\sqrt{n_{1}^{2}\sin{\theta_{in}}^{2}-n_{2}^{2}}},$$

where *k*_0_ is the wavevector in the vacuum and *θ*_in_ is the incident angle in the first medium, assuming it is greater than the critical angle (*θ*_in_ > *θ*_*c*_). The lower limit of the skin depth in isotropic dielectrics (*n* > 1) is $\delta =1/\left(k_{0}\sqrt{n_{1}^{2}-1}\right)$ . Hence, to reduce the skin depth and confine light in the first medium, it is required to use a high-index dielectric as the first medium. However, there is an upper limit for the dielectric constant of transparent dielectrics at optical frequencies, which limits the control of evanescent fields.

When the second medium is anisotropic, another degree of freedom can also control the skin depth [75]:

(2) $$\delta =\frac{1}{k_{0}}\frac{n_{2x}}{n_{2z}}\frac{1}{\sqrt{n_{1}^{2}\sin{\theta_{in}}^{2}-n_{2x}^{2}}},$$

where *n*_2*x*_ and *n*_2*z*_ are the refractive indices of the second medium parallel and perpendicular to the interface, respectively. It is also seen in Eq. (2) that the TIR condition is relaxed to:

(3) $$n_{1}>n_{2x}.$$

We call this new condition relaxed-TIR [76] as it allows us to use the other component of the refractive index independently to control the skin depth. Thus, not only by increasing the contrast between *n*_1_ and *n*_2*x*_, but also by increasing the anisotropy ratio (*n*_2*z*_/*n*_2*x*_ ≫ 1) we can reduce the skin depth [Figure 2(b)] [22], [75]. It is also possible to control the degree of anisotropy in the other way (*n*_2*z*_/*n*_2*x*_ ≪ 1) to extend the penetration of evanescent fields in the second medium [77]. It is worth noting that this skin-depth engineering effect has also been demonstrated in 2D materials exhibiting strong anisotropy [22], [78], [79].

### 3.2 Cylindrical and spherical coordinates

Skin-depth engineering of the TM modes in cylindrical coordinates, when the optical axis is in the *z* direction, is similar to that in the cartesian coordinates [73]. When an isotropic cylinder with a permittivity of *ɛ*_1_ is surrounded by an anisotropic cladding (*ɛ*_2*ρ*_ = *ɛ*_2*φ*_ = *ɛ*_2⊥_ ≠ *ɛ*_2*z*_), the radial wave-vector in the cladding is:

(4) $$k_{2\rho }=\sqrt{\frac{\varepsilon_{2z}}{\varepsilon_{2⊥}}}\sqrt{\beta^{2}-k_{0}^{2}\varepsilon_{2⊥}},$$

where *β* is the wave-vector in the *z* direction (e.g. propagation constant in optical fibers). If *k*_2*ρ*_ becomes imaginary, the field becomes evanescent in the cladding, and by increasing $\sqrt{\varepsilon_{2z}/\varepsilon_{2⊥}}$ , the skin depth can be reduced [73].

When the optical axis is radial, the skin depth engineering in the cylindrical and spherical coordinates becomes more complicated. This is because the anisotropy does not impact the radial wavevector, but it impacts the angular momentum wave number.

In order to analyze this, we consider the wave equations in media exhibiting spherical anisotropy. A similar behavior is observed in cylindrical coordinates [50]. The wave equation for a uniaxial dielectric, with its optical axis aligned along the *r* direction (*ɛ*_*θ*_ = *ɛ*_*φ*_ = *ɛ*_⊥_), can be expressed as [74]:

(5) $$-\frac{1}{\varepsilon_{⊥}}\frac{1}{r^{2}}\frac{\partial }{\partial r}\left(r^{2}\frac{\partial }{\partial r}\left(rE_{r}\right)\right)+\frac{1}{\varepsilon_{r}r^{2}}{\vec{L}}^{2}\left(rE_{r}\right)=k_{0}^{2}\left(rE_{r}\right),$$

where $\hbar \vec{L}=\frac{\hbar }{i}\left(\vec{r}\times \vec{\nabla }\right)$ is the angular momentum operator with an eigenvalue of $\hbar \sqrt{n\left(n+1\right)}$ and *n* is an integer number describing the angular momentum mode number. Note that our focus here is on TM modes, as TE modes remain unaffected by dielectric anisotropy. The first term on the left-hand side of Eq. (5) corresponds to the radial momentum with an eigenvalue of *ℏk*_*r*_ which can be expressed as [74]:

(6) $$\frac{k_{r}^{2}}{\varepsilon_{⊥}}+\frac{n\left(n+1\right)}{\varepsilon_{r}r^{2}}=k_{0}^{2}.$$

The expansion of a plane wave in spherical coordinates with radial anisotropy can be written as [74]:

(7) $$\begin{array}{ll} E_{r}\left(r,\theta ,\varphi \right) & =\frac{1}{{\left(k_{0}r\right)}^{2}}\overset{\infty }{\underset{n=1}{\sum }}c_{n}z_{n_{e}}\left(k_{0}\sqrt{\varepsilon_{⊥}}r\right)P_{n}^{\left(1\right)}\left(\cos\theta \right)e^{\pm i\varphi },\\ n_{e} & =\sqrt{\frac{\varepsilon_{⊥}}{\varepsilon_{r}}n\left(n+1\right)+\frac{1}{4}}-\frac{1}{2}, \end{array}$$

where $P_{n}^{\left(1\right)}$ is the associated Legendre polynomial of the first order, *z*_*n*_ is one of the Riccati–Bessel functions or their superposition, and *c*_*n*_ is the amplitude of the *n*th-mode.

By increasing *n* in Eq. (6), the radial momentum reduces, and when $k_{0}r<\sqrt{n\left(n+1\right)/\varepsilon_{r}}$ , it becomes imaginary. This causes the field to decay faster when it approaches toward the center. The modes with higher angular momentum vanish faster. Increasing *ɛ*_⊥_/*ɛ*_*r*_ leads to a faster decay of evanescent fields. This type of anisotropic media can be utilized as a shell in a core–shell resonator to confine evanescent waves inside the dielectric core and to increase *Q* of the resonators [80], [81], [82].

On the other hand, if we increase the anisotropy in the opposite direction (*ɛ*_⊥_/*ɛ*_*r*_ ≪ 1), near-field evanescent waves can be enhanced without a significant change in the momentum away from the center. The field enhancement using this approach in the subwavelength regime is more substantial than increasing the permittivity in isotropic media [74]. This can lead to a strong conversion of reactive (evanescent) fields near the center into propagating electromagnetic waves even without using metal [50].

A resonator composed of a subwavelength low-index dielectric surrounded by an anisotropic shell (*ɛ*_⊥_/*ɛ*_*r*_ ≪ 1) can enhance the light intensity at the core/shell interface [Figure 2(c) and (d)] due to the enhancement of evanescent fields. The continuity of the displacement current at the boundary also helps to increase the field further without significantly increasing the *Q* [74], [83]. This method can be applied on a chip to demonstrate subwavelength nano-antennas with high scattering efficiency.

## 4 Anisotropic perturbation for crosstalk elimination

Another unique phenomenon that can be observed in a SWG metamaterial-cladded waveguide scheme is anisotropic perturbation. In conventional PICs, two or more parallel dielectric waveguides are often placed in close proximity, resulting in optical coupling between them [59], [84]. This leads to the transfer of optical power from one waveguide to another, causing optical crosstalk. While such optical coupling can be used for various purposes, such as power splitters and polarization controllers, it can also degrade performance in devices like arrayed waveguide gratings (AWGs) [85] and optical phased arrays (OPAs) [86], [87]. To address the crosstalk issue, several approaches have been explored. The most widely used approach involves using waveguide arrays with varying widths (e.g., waveguide superlattice) but requires maintaining inconsistent phases in each channel to reduce crosstalk [88], [89], [90]. Another strategy employs inverse design techniques to minimize crosstalk but at the cost of increased insertion loss and design complexity [91]. Additionally, multimode waveguides can be used in place of multiple single-mode waveguides; however, this approach is still challenged by inter-mode crosstalk, bending loss, and the need for efficient mode (de)multiplexing [92].

**Figure 3:**
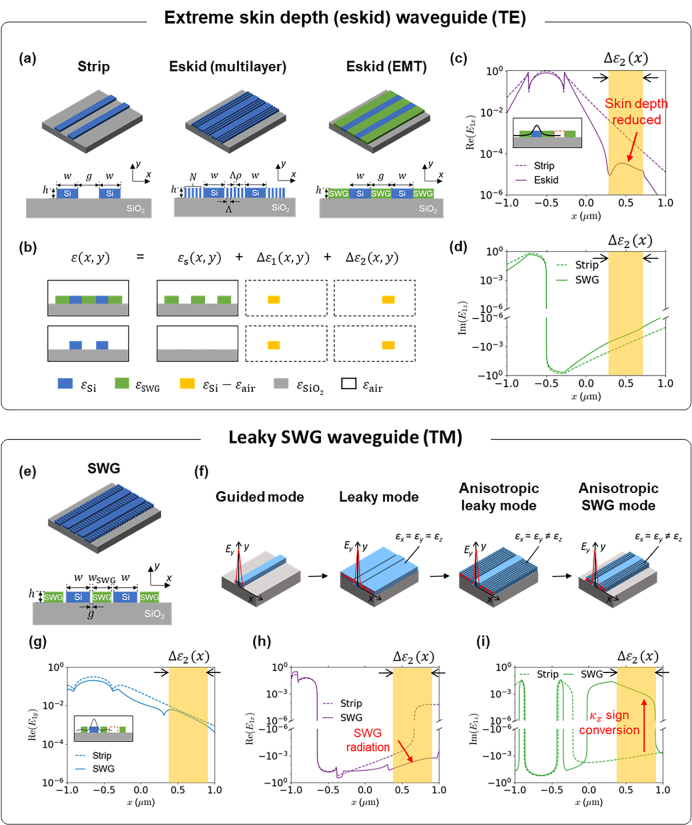
Anisotropic perturbation for zero-crosstalk singularity in coupled waveguides with SWG claddings. (a–d) Extreme skin depth (eskid) waveguides for zero-crosstalk response with TE [94]: (a) Schematic of the coupled strip, eskid (multilayered), and eskid (homogenized equivalent model using EMT) waveguides. (b) Cross-sectional index profile decomposition used to calculate the coupling coefficients with Eq. (8): upper (eskid with EMT) and lower (strip). (c, d) Simulated electric field profiles ***E***_**1**_ at the core center (*y* = *h*/2): (c) Re(*E*_1*x*_) and (d) Im(*E*_1*z*_). The ***E***_**1**_ represents the electric fields with the index profile of *ɛ*_1_ = *ɛ*_s_ + Δ*ɛ*_1_, as shown in the inset. The solid and dashed lines represent eskid and strip waveguides, respectively. The yellow area shows the perturbation region, which determines the coupling coefficient. Note that the eskid significantly suppresses the magnitude of *E*_*x*_ compared to the strip, while its effect on *E*_*z*_ remains low. This anisotropic field suppression is key to achieving zero coupling coefficient, as shown in Figure 4(e). (e–h) Leaky SWG waveguides for zero-crosstalk with TM [95]: (e) schematics of the coupled anisotropic leaky-like SWG waveguides under evaluation. (f) Conceptual evolution of the anisotropic leaky-like mode for TM zero-crosstalk. The anisotropic field oscillations of the leaky wave in the SWG can alter coupling coefficients in *x* and *z* directions to counteract the dominant *E*_*y*_ field. (g–i) Simulated electric field profiles ***E***_**1**_ at the core center (*y* = *h*/2): (g) Re(*E*_1*y*_), (h) Re(*E*_1*x*_), and (i) Im(*E*_1*z*_). The ***E***_**1**_ represents the unperturbed TM modes shown in the inset: anisotropic leaky-like SWG (solid) and strip (dashed) modes. Notice that the non-dominant field intensities and their signs in the perturbation region (yellow-shaded) are changed, allowing anisotropic leaky-like perturbations to achieve zero-crosstalk with TM mode, as shown in Figure 4(i). Detailed perturbation results of each scheme are summarized in Figure 4.

Generally, to minimize optical crosstalk, waveguides must be spaced farther apart, which in turn increases the size of the PIC and fundamentally constrains its integration density. For example, conventional coupled strip waveguides, shown in Figure 3(a), experience significant crosstalk from evanescent field overlap when closely spaced. To mitigate this evanescent field overlap, the concept of skin-depth engineering, as described in Section 3, can be applied by introducing anisotropic cladding. The waveguide scheme that utilizes the SWG configuration in Figure 1(b) as a cladding to suppress skin depth is called an extreme skin-depth (eskid) waveguide. A practical implementation of the eskid waveguide, along with its equivalent model using EMT (shown in green), is also shown in Figure 3(a). The suppression of optical crosstalk using this eskid waveguide scheme was first demonstrated in Ref. [93].

In addition to skin depth engineering, the anisotropic metamaterial cladding introduces an anisotropic perturbation that can completely cancel out the waveguide coupling, resulting in zero-crosstalk. Previously, SWG cladded structures have been explored in the context of multimode interferometers (MMIs) with a large core width [96], where SWGs were used for index contrast engineering rather than for anisotropy-based skin-depth engineering or anisotropic perturbation. Anisotropic perturbation refers to a dielectric perturbation induced by anisotropic media, allowing differential manipulation of coupling coefficients in each direction. The zero-crosstalk response, driven by anisotropic perturbation, arises because the supported channel waveguide modes are either the quasi-TE or quasi-TM mode, having non-negligible field components from all directions (i.e., *E*_*x*_, *E*_*y*_, and *E*_*z*_). For simplicity, we will henceforth refer to these modes as TE and TM, whose dominant fields are *E*_*x*_ and *E*_*y*_, respectively. The coupling coefficient contributed by these non-dominant components can counteract that from the dominant field. Here, the SWG cladding can be engineered to either decrease the coupling strength of the dominant component (as eskid) or increase that of the non-dominant components (as leaky-SWG), making the total coupling coefficient zero. In the following sections, we will summarize and compare anisotropic perturbations realized for both TE [94] and TM [95] modes.

### 4.1 Anisotropic perturbation with eskid waveguide: TE zero-crosstalk

Figure 3(a) illustrates the schematics of the coupled strip and the eskid waveguides. The SWG claddings of the eskid waveguide can be represented as a homogenized metamaterial using the EMT shown in Figure 1. Since this EMT model effectively captures the fundamental impact of the SWG cladding on the coupled eskid waveguides, it will be used for the following analysis. The coupling strength between the waveguides can be quantified by the overall coupling coefficient |*κ*| = |*κ*_*x*_ + *κ*_*y*_ + *κ*_*z*_|, where each component *κ*_*i*_ (*i* = *x*, *y*, and *z*) is calculated by [59], [97]:

(8) $$\kappa_{i}=\frac{\omega \varepsilon_{0}}{4}\iint E_{1i}\left(x,y\right)\Delta \varepsilon \left(x,y\right){E_{2i}}^{*}\left(x,y\right)dxdy.$$

Here, *E*_1*i*_(*x*, *y*) and *E*_2*i*_(*x*, *y*) represent the *i*-components of the normalized mode profiles for unperturbed waveguide modes 1 and 2, respectively, and Δ*ɛ*(*x*, *y*) denotes the perturbation index. To calculate Eq. (8), the index distribution *ɛ*(*x*, *y*) of the coupled eskid waveguides can be decomposed as shown in Figure 3(b): the static index *ɛ*_s_(*x*, *y*) representing the background (i.e., except the core regions), and the perturbation indices Δ*ɛ*_1_(*x*, *y*) and Δ*ɛ*_2_(*x*, *y*) due to the presence of each waveguide. The normalized field profiles ***E***_**1**_(*x*, *y*) and ***E***_**2**_(*x*, *y*) used in Eq. (8) are calculated with the index profiles *ɛ*_1_(*x*,s *y*) = *ɛ*_s_(*x*, *y*) + Δ*ɛ*_1_(*x*, *y*) and *ɛ*_2_(*x*, *y*) = *ɛ*_s_(*x*, *y*) + Δ*ɛ*_2_(*x*, *y*), respectively. The key factor in determining the coupling coefficient is the field interaction within the perturbation region. For example, if we set the waveguide 2 (right-side) as the perturbation (i.e., Δ*ɛ* = Δ*ɛ*_2_), then the interaction between the evanescent field of *E*_1*i*_ and the confined field of *E*_2*i*_ determines *κ*_*i*_. The sign of the coupling coefficient can be either positive or negative, depending on the field distributions of *E*_1*i*_ and *E*_2*i*_ in this perturbation region. By placing anisotropic SWG cladding [Figure 1(b)], the evanescent field can be engineered anisotropically, manipulating the coupling coefficient to achieve even *κ* = 0; this is the key idea behind anisotropic perturbation to achieve a zero-crosstalk.

**Figure 4:**
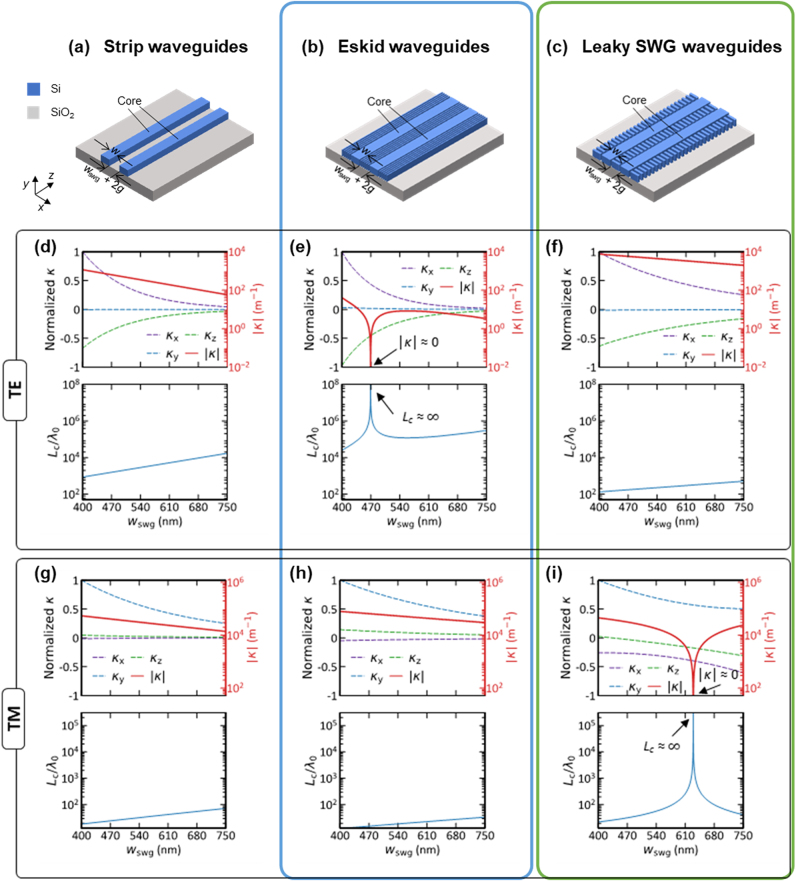
Dielectric perturbation and coupling length comparisons between waveguides with different anisotropic claddings for TE and TM [94], [95]. Dielectric perturbation coefficients are plotted to illustrate the coupling mechanisms. (a–c) Schematics of the coupled strip, eskid, and leaky-SWG waveguides, respectively. (d–i) Normalized coupling coefficients *κ*_*x*_ (purple dashed), *κ*_*y*_ (blue dashed), and *κ*_*z*_ (green dashed), along with the overall coupling coefficient |*κ*| = |*κ*_*x*_ + *κ*_*y*_ + *κ*_*z*_| (red solid), are plotted as a function of SWG width *w*_swg_. The corresponding normalized coupling length *L*_c_/*λ*_0_ (solid blue line) is shown below in each plot. (d–i) Represent TE and TM polarizations, respectively, for each configuration: (d, g) strip, (e, h) eskid, and (f, i) leaky-SWG waveguides. The effective medium theory is used to model the *ɛ*_‖_ and *ɛ*_⊥_ in (b) and (c) schemes. Note that, for TE in the eskid (e), the overall |*κ*| approaches zero, and the coupling length increases to infinite, due to anisotropically suppressed field perturbations as shown in Figure 3(c) and (d). Similarly, for TM in the leaky-SWG (i), the |*κ*| becomes zero, resulting in an infinite coupling length, but due to anisotropic leaky-like oscillations as shown in Figure 3(g) and (h). Geometric parameters are height *h* = 220 nm, core width *w*_0_ = 530 nm, and *g* = 65 nm, with *ρ* = 0.45, and a wavelength of *λ*_0_ = 1,550 nm. Reproduced with permission from Ref. [94], [95].

Figure 3(c) and (d) respectively plot the real *E*_1*x*_ and the imaginary *E*_1*z*_ of the TE mode in eskid (solid) and strip (dashed), along the *x*-axis (at *y* = *h*/2). The contribution of *E*_1*y*_ is negligible. The inset illustrates the simulated index profile *ɛ*_1_(*x*, *y*). The yellow-shaded region highlights the perturbation region that determines the coupling coefficient. As expected, in Figure 3(c), the skin depth of *E*_1*x*_ is significantly reduced in the eskid configuration compared to the strip, resulting in a lower *κ*_*x*_ for the eskid waveguide. However, in Figure 3(d), the eskid has a smaller effect on *E*_1*z*_ compared to *E*_1*x*_, resulting in a comparable |*κ*_*z*_| as in strip. This anisotropic skin depth effect reduces the difference in magnitude between *κ*_*x*_ and *κ*_*z*_, and since these coupling coefficients have opposite signs, the overall coupling coefficient |*κ*| can be minimized or even reduced to zero by tailoring the geometric parameters. The detailed results are shown in Figure 4(e).

### 4.2 Anisotropic perturbation with leaky-SWG: TM zero-crosstalk

The idea of skin depth suppression used in the eskid waveguide is effective only for TE. For TM, the eskid would rather increase the crosstalk. Unlike in TE, the coupling coefficients of a guided TM mode do not have the negative *κ*_*i*_ terms that are essential for anisotropic perturbation. To achieve the anisotropic perturbation in TM, a waveguide design with SWG claddings aligned along the propagation direction was introduced, as shown in Figure 3(e) [95]. In this scheme, minor coupling coefficients *κ*_*x*_ and *κ*_*z*_ emerge due to oscillating field behavior within the cladding region. This oscillation is caused by the radiative leaky mode, which generally leads to strong coupling between waveguides, even at a far distance, when the cladding materials are isotropic [98], [99]. However, with anisotropic SWGs, the oscillatory behavior of each field component can be controlled anisotropically. This control enables the minor coupling coefficients *κ*_*x*_ and *κ*_*z*_ to counteract and compensate for the dominant coupling coefficient *κ*_*y*_, thereby reducing the overall coupling.

Figure 3(f) illustrates the conceptual formation of the anisotropic leaky-like SWG mode. Figure 3(g)–(i) plot the Re(*E*_1*y*_), Re(*E*_1*x*_), and Im(*E*_1*z*_) for both leaky-SWG (solid) and strip (dashed) configurations along the *x* axis (at *y* = *h*/2). Similar to the TE, the inset shows the simulated index profile *ɛ*_1_(*x*, *y*), with the yellow-shaded region indicating the perturbation region Δ*ɛ*_2_. For the dominant field Re(*E*_1*y*_), the difference between leaky-SWG and strip configurations is minimal [Figure 3(g)]. However, the magnitude of *E*_1*x*_ in the leaky-SWG significantly increases in the perturbation region compared to the strip, leading to a non-negligible *κ*_*x*_, which counterbalances the *κ*_*y*_ [Figure 3(h)]. For the *z*-component, the sign of *E*_1*z*_ is reversed in the perturbation region, becoming opposite to that of *E*_2*z*_, resulting in an overall negative *κ*_*z*_ [Figure 3(i)]. As a whole, the SWGs claddings induce negative *κ*_*x*_ and *κ*_*z*_ that counteract the positive *κ*_*y*_, reducing the overall coupling coefficient even in the presence of a radiative leaky mode. By optimizing the geometric parameters, further tuning can achieve zero-|*κ*|, as in Figure 4(i).

### 4.3 Coupling length comparison

The coupling coefficients and the corresponding coupling length *L*_*c*_/*λ*_0_ of the strip, eskid, and leaky-SWG waveguides are summarized and compared in Figure 4. Figure 4(a)–(c) illustrate the schematics of the coupled strip, eskid, and leaky-SWG waveguides, respectively. Figure 4(d)–(i) plot the simulated coupling coefficients and *L*_*c*_/*λ*_0_ for TE and TM modes, respectively, as a function of SWG width *w*_swg_. In these figures, the normalized *κ*_*x*_, *κ*_*y*_, and *κ*_*z*_ are represented by purple, blue, and green dashed lines (left axis), while the magnitude of the total coupling coefficient |*κ*| is shown as a red solid line (right axis). The corresponding coupling length is calculated by *L*_c_ = *π*/(2|*κ*|) and plotted below with a solid blue line. All SWGs are modeled using the EMTs.

For TE mode, where *κ*_*x*_ is dominant [Figure 4(d)–(f)], *κ*_*y*_ is negligible and *κ*_*z*_ is negative in all configurations. The negative *κ*_*z*_ reduces the coupling due to *κ*_*x*_. In all configurations, the coupling coefficients decrease as *w*_swg_ increases due to the reduced field overlap, leading to longer coupling lengths. In a typical strip waveguide, *κ*_*x*_ is dominant, resulting in a finite coupling length [Figure 4(d)]. However, in the eskid, skin depth suppression significantly reduces |*κ*_*x*_|, as shown by the coupling length plot. In general, the *L*_*c*_/*λ*_0_ of the eskid [Figure 4(e)] is approximately one order of magnitude longer than that of the strip waveguide [Figure 4(d)]. Furthermore, at a specific SWG width (*w*_swg_ ≈ 470 nm), *κ*_*z*_ completely cancels out the *κ*_*x*_, making the overall coupling coefficient zero (*κ* = 0). This corresponds to an infinitely long coupling length, i.e., *L*_c_ = ∞. In contrast, for the leaky-SWG configuration, the anisotropic cladding indices *ɛ*_*x*_ and *ɛ*_*z*_ are reversed, extending the evanescent wave and causing larger crosstalk [Figure 4(f)]. Although this configuration is not ideal for low crosstalk, it can be used for an efficient coupler with a shorter coupling length [73], [77].

For TM mode, where *κ*_*y*_ is dominant [Figure 4(g)–(i)], other *κ*_*x*_ and *κ*_*z*_ are relatively negligible in guided modes, as shown in Figure 4(g) and (h). Only the leaky-SWG configuration exhibits non-negligible *κ*_*x*_ and *κ*_*z*_ [Figure 4(i)], due to the oscillatory field pattern in the cladding associated with the leaky radiation. The *κ*_*x*_ and *κ*_*z*_ are negative due to the field overlaps shown in Figure 3(h) and (i). Unlike the other guided modes, the magnitudes of *κ*_*x*_ and *κ*_*z*_ increase as *w*_swg_ enlarges due to the oscillating field nature in the cladding. This allows for the zero total coupling coefficient |*κ*| = 0, leading to an infinitely long coupling length *L*_c_ = ∞. Here, this is achieved near *w*_swg_ ≈ 630 nm. On the other hand, the eskid generates positive *κ*_*z*_, resulting in a higher |*κ*| and a shorter coupling length [Figure 4(h)].

### 4.4 Experimental demonstrations of zero-crosstalks in TE and TM

**Figure 5:**
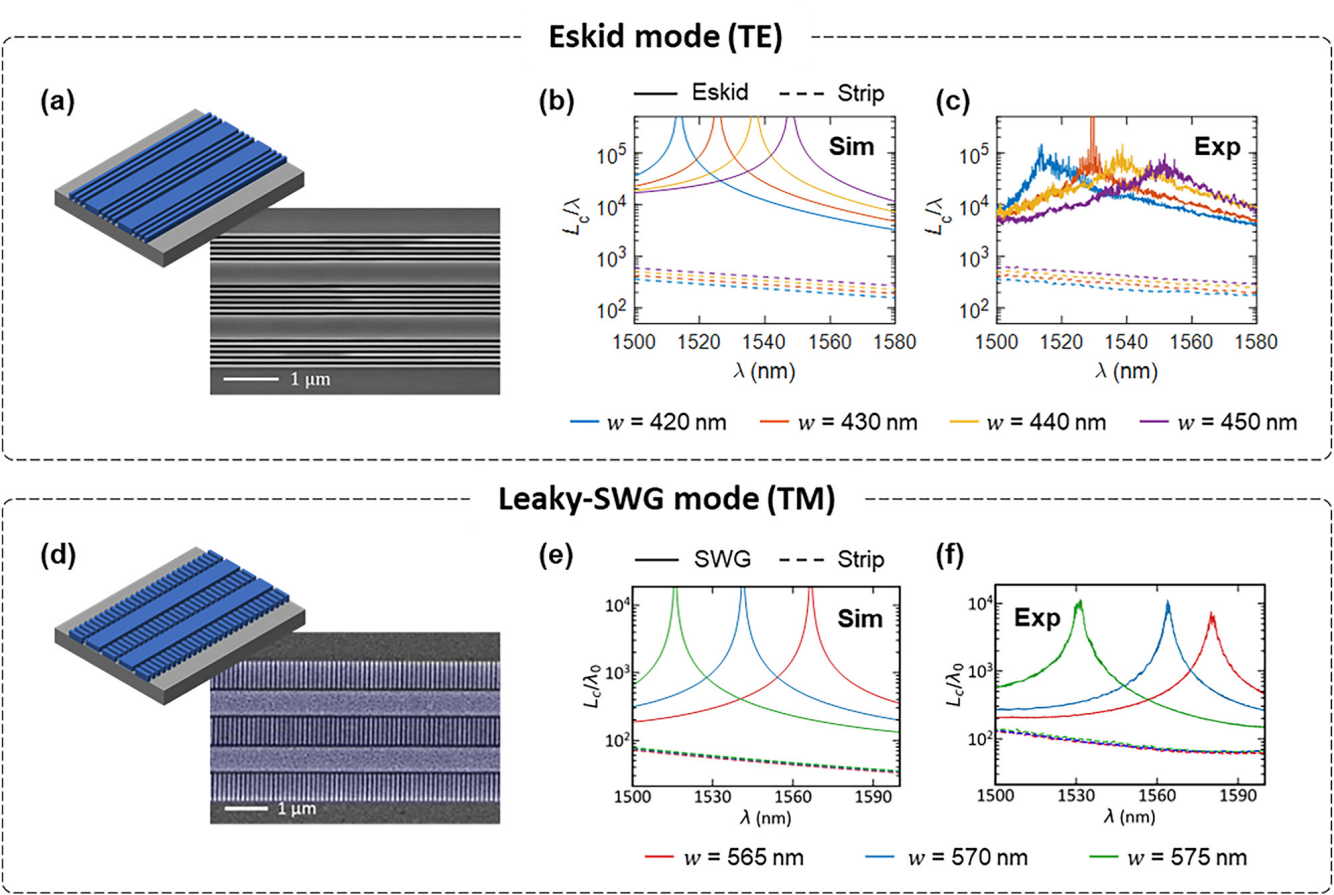
Experimental demonstrations of zero-crosstalk singularity in the eskid and leaky-SWG waveguides. (a–c) Eskid waveguide with TE mode [94]; (d–f) leaky-SWG waveguide with TM mode [95]. Schematics and SEM images of the fabricated (a) eskid and (d) leaky-SWG waveguides. (b) Simulated and (c) experimentally characterized coupling lengths *L*_c_/*λ*_0_ as functions of free-space wavelength *λ*_0_ for different waveguide widths: *w* = 420 nm (blue), *w* = 430 nm (red), *w* = 440 nm (yellow), and *w* = 450 nm (purple). Solid and dashed lines represent the eskid and strip waveguides, respectively. (e) Simulated and (f) experimentally characterized *L*_c_/*λ*_0_ as functions of *λ*_0_ for different waveguide widths: *w* = 565 nm (red), *w* = 570 nm (blue), and *w* = 575 nm (green), with solid and dashed lines representing the leaky-SWG and strip waveguides, respectively. The peaks in *L*_c_/*λ*_0_ represent zero-crosstalk singularities with infinitely long coupling lengths. Reproduced with permission from Refs. [94], [95].

The zero-crosstalk responses in TE and TM modes, as discussed in Section 4.3, has been experimentally demonstrated. Figure 5(a)–(d) show the schematic and SEM images of the fabricated devices, with SWG arrays parallel (eskid) and perpendicular (leaky-SWG) to the propagation direction, respectively. The output power ratio *I*_2_/*I*_1_, which defines optical crosstalk, was measured by sending light through one of the coupled waveguides. The coupling length *L*_c_ was then extracted using the following relation [59]:

(9) $$\frac{I_{2}}{I_{1}}=\tan^{2}\left(\frac{\pi L}{2L_{\text{c}}}\right),$$

where *L* is the physical length of the coupled waveguides, and *I*_1_ and *I*_2_ are output powers at the through and coupled ports, respectively.

For TE mode, Figure 5(b) and (c) show the *L*_c_/*λ* spectra of coupled eskid (solid) and strip (dashed) waveguides: (b) simulations and (c) experiments. Each color represents a different core width, while fixing the *w*_swg_. In conventional coupled strip waveguides (dashed lines), the coupling lengths are less than $\approx 10^{3}$ wavelengths, regardless of core width *w*. However, in the coupled eskid waveguides (solid lines), *L*_c_/*λ* is extended by one to two orders of magnitude, primarily due to the reduced skin-depth effect. In addition, depending on the core width, a coupling length singularity appears where *L*_*c*_/*λ* = ∞, originating from the anisotropic perturbation effect. In the experiment, the peak *L*_c_/*λ* observed is around 10^5^, which is likely limited by detector sensitivity or background noise of the system. Nevertheless, the significant increase in coupling length is clearly shown, demonstrating the longest coupling length.

Similarly, Figure 5(e) and (f) show the *L*_c_/*λ* spectra for the coupled leaky-SWG waveguides with TM mode. Due to the lower confinement of TM compared to TE, the coupling lengths of the strip (dashed lines) are less than 10^2^ wavelengths. However, in the leaky-SWG configuration, *L*_c_/*λ* can be increased by approximately 10^4^ wavelengths, though the experimental results are again limited by the measurement system. Since the anisotropic perturbation is sensitive to geometric parameters, the corresponding spectrum also exhibits high sensitivity. This feature makes the leaky-SWG configuration particularly suitable for applications such as refractive index sensors [100]. Further optimization through dispersion engineering or tapering could help reduce the spectral sensitivity and broader bandwidth, providing more stable device performance.

## 5 Applications of anisotropic SWG metamaterials in PICs

**Figure 6:**
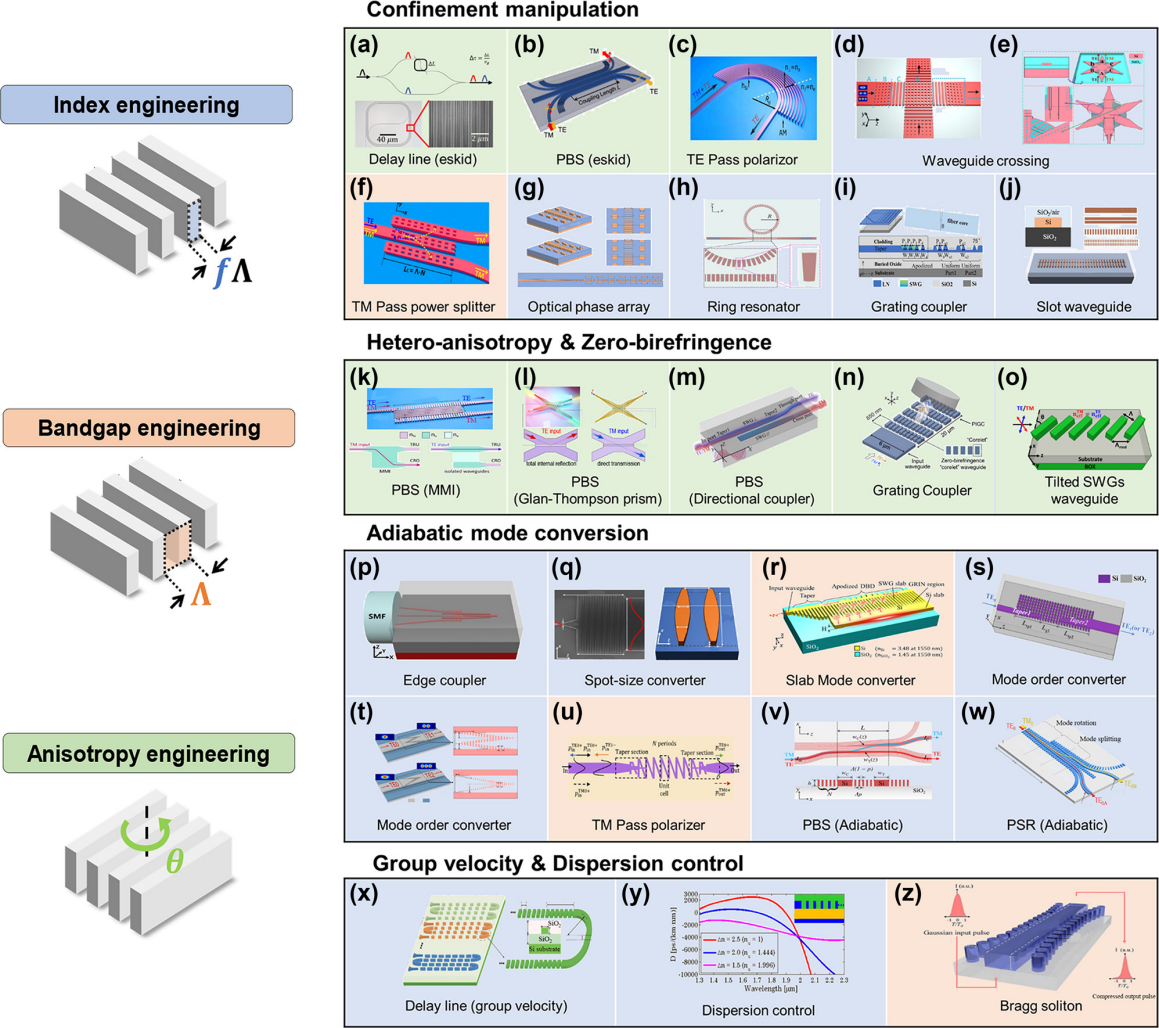
Component-level applications of anisotropic SWG metamaterials in PICs. SWG anisotropic metamaterials can introduce three types of engineering: index (blue-shaded), bandgap (orange-shaded), and anisotropy (green-shaded) engineering. These approaches, applied independently or in combination, enhance various modal characteristics: (a–j) confinement manipulation, (k–o) hetero-anisotropy and zero-birefringence, (p–w) adiabatic mode conversion, and (x–z) group velocity and dispersion control. Confinement manipulation: (a) dense delay-line using eskid waveguide [101], (b) high extinction ratio polarization beam splitter (PBS) with eskid waveguide [102], (c) TE pass polarizer with SWG claddings [103], (d) multimode waveguide crossing with SWGs [47], (e) 3 × 3 star-crossing waveguide with bent-SWGs [104], (f) TM-pass polarizer with holey-SWGs [105], (g) optical phased array with SWG tapers [106], (h) refractive index sensing micro-ring resonator with trapezoidal SWGs [107], (i) grating coupler with apodized SWGs [108], and (j) slot SWG waveguide [109]. Hetero-anisotropy and zero-birefringence: broadband PBSs based on (k) SWGs multi-mode interferometer [110], (l) SWGs Glan–Thompson prism [111], (m) SWGs directional coupler [112], polarization-independent (n) grating coupler [113], and (o) tilted SWG waveguide [114]. Adiabatic mode conversion: (p) edge couplers with SWG cores [115], (q) spot-size converter using SWGs GRIN lens [55], (r) slab mode converter using apodized Bragg deflectors and graded-index waveguide [56], (s) mode-order converters using bricked SWGs [116], (t) polygonal SWG phase shifter [117], (u) TM-pass polarizer with tilted SWGs [118], and (v) broadband PBS [119] and (w) polarization splitter-rotator with SWG claddings [120]. Group delay and dispersion control: (x) delay line with variable group velocity via SWG filling fraction control [121], (y) dispersion control using SWGs [122], and (z) Bragg solitons with cladding-modulated grating [123]. Reproduced with permission from Refs. [55], [56], [47], [101], [102], [103], [104], [105], [106], [107], [108], [109], [110], [111], [112], [113], [114], [115], [116], [117], [118], [119], [120], [121], [122], [123].

We have introduced two fundamental physics uniquely enabled by anisotropic metamaterials: skin-depth engineering (Section 3) and anisotropic perturbation (Section 4). These are easy to implement using SWG metamaterials, though their applications are not limited to dense integration. While these fundamentals may not always be explicitly highlighted, they underpin most component-level applications of SWG metamaterials in a broader context. In this section, we present a comprehensive overview of component-level PIC applications that leverage anisotropic SWG metamaterials. Figure 6 illustrates the recent state-of-the-art SWG metamaterial devices, which are categorized based on three key engineering techniques: Index (blue-shaded), bandgap (orange-shaded), and anisotropy (green-shaded) engineering. Each approach can be fine-tuned by adjusting the geometric parameters of SWGs. For example, the SWG filling fraction *f* can control the effective indices (index engineering); the pitch size Λ can translate the wave band regimes between the SWG, Bragg, and radiation (bandgap engineering); and, the SWG angle *θ* directly manipulates the anisotropy (anisotropy engineering). It is important to note that these three approaches are interrelated, with each affecting the others to some extent. Nevertheless, we categorize the devices based on the dominant effect of each technique, and the SWG devices discussed here have utilized one or more of these engineering strategies, enabling precise and efficient control of light in PICs. We further categorized each PIC component based on four distinct device functions: confinement manipulation, hetero-anisotropy/zero-birefringence, adiabatic mode conversion, and group velocity and dispersion control, emphasizing the critical roles that SWG metamaterials play in enabling/enhancing devices’ functionalities. In the following subsections, we will explore the primary functions of SWG metamaterials within the context of each of these principles. Given the extensive works of SWG-enhanced PIC components, a comprehensive discussion is beyond the scope of this review. Readers seeking a broader overview of foundational SWG research are referred to existing review articles [27], [28], [29], [30], [31].

### 5.1 Confinement manipulation

The fundamental basis of a PIC is the waveguide mode, and manipulating its confinement by engineering the core/cladding indices (and thus the index contrast) and their anisotropy plays a crucial role in overall device performance. Each component may require either high or low confinement, depending on its specific functionality. Here, the SWGs, with their index modulation effect and anisotropic characteristics as discussed in previous sections, significantly expand the range of confinement control. This flexibility has made SWGs a key tool for tailoring confinement engineering.

For example, the zero-crosstalk response introduced in Section 4 can be directly employed for a densely packed delay line [Figure 6(a)] [101] and a highly selective polarization beam splitter (PBS) [Figure 6(b)] [102]. In all these cases, the tight field confinement enabled by the anisotropic nature of SWGs is leveraged to enhance device performance. This tight confinement also helps reduce bending loss by suppressing radiation losses, making it effective even in high-radiative-loss regimes where small bending radii are required [93], [124]. This enables the design of a compact TE pass polarizer, where only the TE mode is highly confined with reduced bending loss [Figure 6(c)] [103]. When integrated into both the core and cladding, SWGs allow greater flexibility in adjusting index contrast and anisotropy, thereby improving confinement control [125]. A waveguide crossing bar is another good example. In Figure 6(d) [47], 1D and 2D SWGs are combined to create a compact crossing bar while effectively suppressing unwanted side couplings. The 1D SWG acts as an anisotropic cladding, reducing the skin depth to prevent coupling to unintended ports, while the 2D SWGs facilitate smooth modal transitions, minimizing reflections from the 1D SWG regions. Alternatively, more complex SWG designs, such as nanohole SWGs and fan-shaped bent SWGs, can be used to manipulate the modal phase and index distribution, further enhancing the focusing of multimodal interference crossing bars [Figure 6(e)] [104].

Conversely, reducing the index contrast between the core and cladding allows light to leak into the cladding, extending the evanescent field and making it easier to couple light into other structures. These characteristics allow for rapid light transitions to desired locations. For example, in Figure 6(f) [105], the TM-pass splitter functions as a Bragg reflector for TE while acting as a directional coupler for TM. The SWGs in this design are optimized to create strong evanescent coupling for TM, enabling fast power transitions. Similarly, in the optical phased array shown in Figure 6(g) [106], the core index is reduced using SWGs, which enhances coupling to adjacent emitters and improves radiation efficiency for beam steering.

SWGs can also modulate modal field distributions and exploit optical properties, beyond merely adjusting light confinement. For example, by fabricating trapezoidal SWGs with different duty cycles, light can be biased towards the larger duty cycle, resulting in an asymmetric light distribution. This can be used to achieve faster light coupling in the power splitter [126] or to concentrate light within the inner side of a ring resonator, reducing bending loss and improving the quality factor [Figure 6(h)] [107]. Additionally, apodizing the filling fraction of SWGs can modulate the diffraction levels, creating a Gaussian mode distribution that enhances coupling efficiency [Figure 6(i)] [108]. SWGs can also be used to form and modulate a slot mode, which strongly confines light in the middle of a low-index region [Figure 6(j)] [109], [127].

### 5.2 Hetero-anisotropy and zero-birefringence

One of the most distinctive properties that SWGs offer is their ability to control anisotropy. The anisotropy in a structure can either be uniformly distributed or vary across different regions, which is referred to as hetero-anisotropy. The anisotropy of SWGs can be tailored by adjusting the filling fractions, orientations, and shapes of the gratings, making them ideal for implementing and manipulating hetero-anisotropy in PICs. SWGs can manipulate TE and TM modes differently within the same structure, a feature that is effectively applied in polarization handling devices such as PBSs and polarization splitters and rotators (PSRs).

Extensive research has led to the development of advanced SWG-supported PBSs that leverage hetero-anisotropy for enhanced performance. For example, the PBS shown in Figure 6(k) confines the TE to the upper waveguide due to the reduced skin depth caused by anisotropy, while the TM passes to the lower waveguide through a multimode interference (MMI) [110]. The inclusion of anisotropic SWGs can broaden the MMI bandwidth [42], [128] and further optimization is achieved by tilting the SWGs [129]. Similarly, in Figure 6(l), TM propagates through a homogeneous material to the lower port without experiencing total internal reflection (TIR), while TE undergoes TIR and is directed to the upper port [111]. This design, based on the Glan–Thompson prism, uses anisotropic cement to reduce the skin depth and enhance TE confinement, improving polarization selectivity. In Figure 6(m), two orthogonal SWG core waveguides are coupled, forming an efficient directional coupler for the TM and a low-crosstalk waveguide for the TE, thus effectively enabling polarization splitting [112].

While hetero-anisotropy is often used to enhance anisotropic behavior, SWGs can also be engineered to reduce modal anisotropy and achieve zero-birefringence. Generally, in PICs, the waveguide width is larger than the height for easier etching during fabrication. This leads to a higher effective index for TE than TM, resulting in significant birefringence and polarization sensitivity. However, this issue can be addressed by carefully engineering the anisotropy of SWGs, making it possible to equalize the modal indices for both TE and TM modes. For example, when SWGs are placed parallel to the propagation direction [as in Figure 1(b)], the permittivity in the TE direction (*ɛ*_*x*_ = *ɛ*_⊥_) is lower than that in the TM direction (*ɛ*_*y*_ = *ɛ*_‖_), since *ɛ*_⊥_ < *ɛ*_‖_. Therefore, by tuning the duty cycle, it is possible to equalize the refractive indices *ɛ*_*x*_ and *ɛ*_*y*_ equal, even in structures with asymmetric height-to-width aspect ratio. Similarly, zero-birefringence can also be achieved using 2D SWG patterns [Figure 6(n)]. This approach was adopted for the development of polarization-independent grating couplers [48], [113]. Moreover, alternatively, tilting the SWGs at a specific angle can achieve a zero-birefringence waveguide, as in Figure 6(o) [114]. While the TM mode’s modal index remains largely unaffected by SWG tilt, the TE mode’s index varies significantly with the tilt angle, enabling TE and TM index matching through angle optimization.

### 5.3 Adiabatic mode conversion

In many PIC components, adiabatic mode conversion is widely used to minimize losses caused by abrupt modal transitions. This can be achieved through gradual structural changes, which prevent discontinuities and maintain a continuous state. Such smooth transition between modes also allows for a broader bandwidth, improving fabrication tolerance. A simple method for adiabatic mode conversion is geometric tapering, where the index distribution is adjusted gradually along the propagation direction, enabling efficient mode transition with high conversion efficiency. However, the gradual transition often leads to a larger footprint, limiting the overall compactness of devices. Here, SWGs provide extra degrees of freedom for efficient modal transitions, for example, by allowing index redistribution through adjustments in the filling fraction. SWGs are often combined with geometric tapering to enhance modal conversion efficiency and minimize device size [130], [131], [132], [133]. Tuning the duty cycle of SWGs also can lower the index, simplifying the design and making it more tolerant to fabrication errors.

A widely explored application of this approach is a fiber-to-chip edge coupler [24], [25], [26], [52], [53], [54]. Edge couplers generally offer broader bandwidth than grating couplers but have larger footprints due to the tapering required for mode transition and are sensitive to fiber alignment with small mode field diameter (MFD) [134], [135]. By using SWGs as a core and adjusting their filling fraction, faster adiabatic transition can be achieved, resulting in more compact designs [Figure 6(p)] [115]. Moreover, using SWGs in the cladding with a lower filling fraction can reduce the index contrast, thereby increasing the MFD and improving alignment tolerance [54]. Numerous innovative SWG-based edge couplers have been explored extensively [52], [53], [136], [137].

Another key advantage of SWGs is their flexibility in shape and orientation, allowing for the creation of metamaterials with diverse properties beyond simple perpendicular or parallel arrangement. For example, gradient-index (GRIN) metalenses can be easily implemented by spatially varying the filling fractions of SWGs in different shapes. GRIN structures are particularly effective at expanding the mode size, making them ideal for applications such as spot size converter [Figure 6(q)] [55] and slab mode converter [Figure 6(r)] [56], achieving large modal conversion within a compact footprint. Various SWG shapes have been developed, including bricked SWGs for efficient mode-order transitions from fundamental TE to higher-order TE_1_ and TE_2_ modes [Figure 6(s)] [116]; polygonal SWGs for simultaneous phase and amplitude control to enable rapid mode conversion [Figure 6(t)] [117]; and *N*-shape tapered SWGs for a TM-pass polarizer, which enables selective TM mode transition while reflecting the TE mode in the Bragg regime [Figure 6(u)] [118].

Although only a few applications are introduced here, adiabatic mode conversion can be integrated into nearly all PIC components, often combined with other functions. Notable examples include adiabatic PBS [Figure 6(v)] [119] and PSR [Figure 6(w)] [120], where the anisotropic properties of SWGs, combined with adiabatic tapering, significantly enhance device performance. By leveraging these adiabatic mode transitions, SWGs enable efficient mode transitions, broader bandwidth, and improved fabrication tolerance.

### 5.4 Group velocity and dispersion control

Group velocity (*v*_*g*_) represents the speed at which the energy or information within the envelope of a wave propagates through a medium. Since group velocity strongly depends on modal confinement and wavelength dependency of effective indices, it can be precisely fine-tuned using the index engineering of SWGs. This tuning is essential for optimizing delay lines, managing dispersion and pulse propagation, and enhancing nonlinear optical processes.

For example, delay times in a delay line can be controlled by varying the filling fractions of SWGs rather than adjusting physical waveguide lengths. Traditional delay lines achieve longer delay times by increasing waveguide length, which typically leads to a larger footprint and increased signal loss. SWG-based delay lines, however, enable group velocity adjustment without changing length, resulting in more compact designs [Figure 6(x)] [121].

Index engineering of SWGs also provides control over modal dispersion [122]. Generally, waveguide modes with higher confinement exhibit anomalous dispersion, whereas lower confinement results in normal dispersion. By using SWGs to adjust modal confinement, a broad range of dispersion profiles can be synthesized [Figure 6(y)], which can be applied to optical communications, dispersion compensation, and optical signal processing.

Additionally, manipulating group velocity and dispersion through SWGs can significantly enhance nonlinear optical processes. One prominent example is four-wave mixing, a nonlinear interaction in which two or more waves combine to produce new optical frequencies. Achieving broadband phase matching in this process is challenging, but engineering SWGs to create a graded index profile allows different spatial modes with equidistant frequencies and a common wavevector, thus enabling broadband phase matching [138]. Another compelling nonlinear application is the generation of Bragg solitons through bandgap engineering of SWGs [123]. Near the band edge, strong anomalous dispersion can induce soliton compression, allowing on-chip Bragg soliton generation through precise tuning of the SWG grating pitch [Figure 6(z)]. Incorporating SWGs into the air cladding also allows for dispersion engineering that supports dispersive waveguide in supercontinuum generation [139].

## 6 Summary and outlook

SWGs on integrated photonic platforms, particularly with anisotropic characteristics, have revolutionized the field of nanophotonics by offering unprecedented control over modal properties. Their ability to manipulate skin depth and anisotropic perturbation has led to the development of efficient, low-crosstalk, and compact photonic devices, promising scalable and high-density PICs. This review explored a broad range of applications of anisotropic SWG metamaterials, categorized into four functional types: confinement manipulation, hetero-anisotropy and zero-birefringence, adiabatic mode conversion, and group velocity and dispersion control. We also highlighted the key engineering methodologies behind each device, namely index, bandgap, and anisotropy engineering.

Although substantial advancements have been achieved, several unexplored questions still remain to be addressed. While much of the progress thus far has focused on modal engineering using SWG configurations aligned parallel or perpendicular to the wave propagation, alternative designs, such as tilted and bianisotropic metamaterials, hold promise for further innovation. These configurations could unlock new methods of controlling waveguide couplings, particularly in platforms with lower index contrasts or ridge-type waveguides, where skin depth effects are limited. As we continue to push the limits of chip density and miniaturization, new approaches will find opportunities for the next generation of photonic devices.

In addition to reducing modal size and suppressing crosstalk, further research could benefit from expanding mode size to interface with free-space modes in more diverse ways. Though some work has been done to increase mode size using SWGs, the impact of anisotropy in this context is relatively underexplored and could be crucial. Moreover, the effect of gain and loss on skin depth and anisotropic perturbation remains largely unexplored, presenting an opportunity to investigate how these factors affect the overall performance of SWG-based cavities and lasers. Understanding the modulation of gain and loss in these systems could lead to more precise control of light propagation, confinement, and amplification. Furthermore, the direct interplay between TE and TM can be fully addressed using anisotropic metamaterials, which opens the door for devices’ polarization (in)sensitivity and other novel functionalities. By integrating anisotropic metamaterials with active media, it may be possible to realize new dimensions of active light manipulation and unlock unprecedented capabilities.

To fully capitalize on the full potential of anisotropic metamaterials, it will be crucial to integrate these approaches into CMOS-compatible processes. Early demonstrations have shown that skin-depth engineering can be implemented using CMOS technology [77], [124], but achieving higher density and finer control will require advancements in photolithographic resolution. Furthermore, integrating SWGs into other photonic platforms beyond silicon-on-insulator, such as silicon nitride and lithium niobate, offers additional pathways for expanding the application space and performance versatility of PICs. As these processes mature, the potential for scaling up photonic chip integration using anisotropic metamaterials becomes even more compelling.

In conclusion, anisotropic metamaterials have already proven their value in advancing PIC technology, and the next phase of research will likely bring transformative breakthroughs as a whole system. By further refining anisotropic control, researchers can unlock new device architectures and significantly enhance the scalability of PICs, dramatically increasing the number of components on a single PIC chip. We envision that the SWG-integrated PICs will play a pivotal role in shaping the future of photonic platforms.
