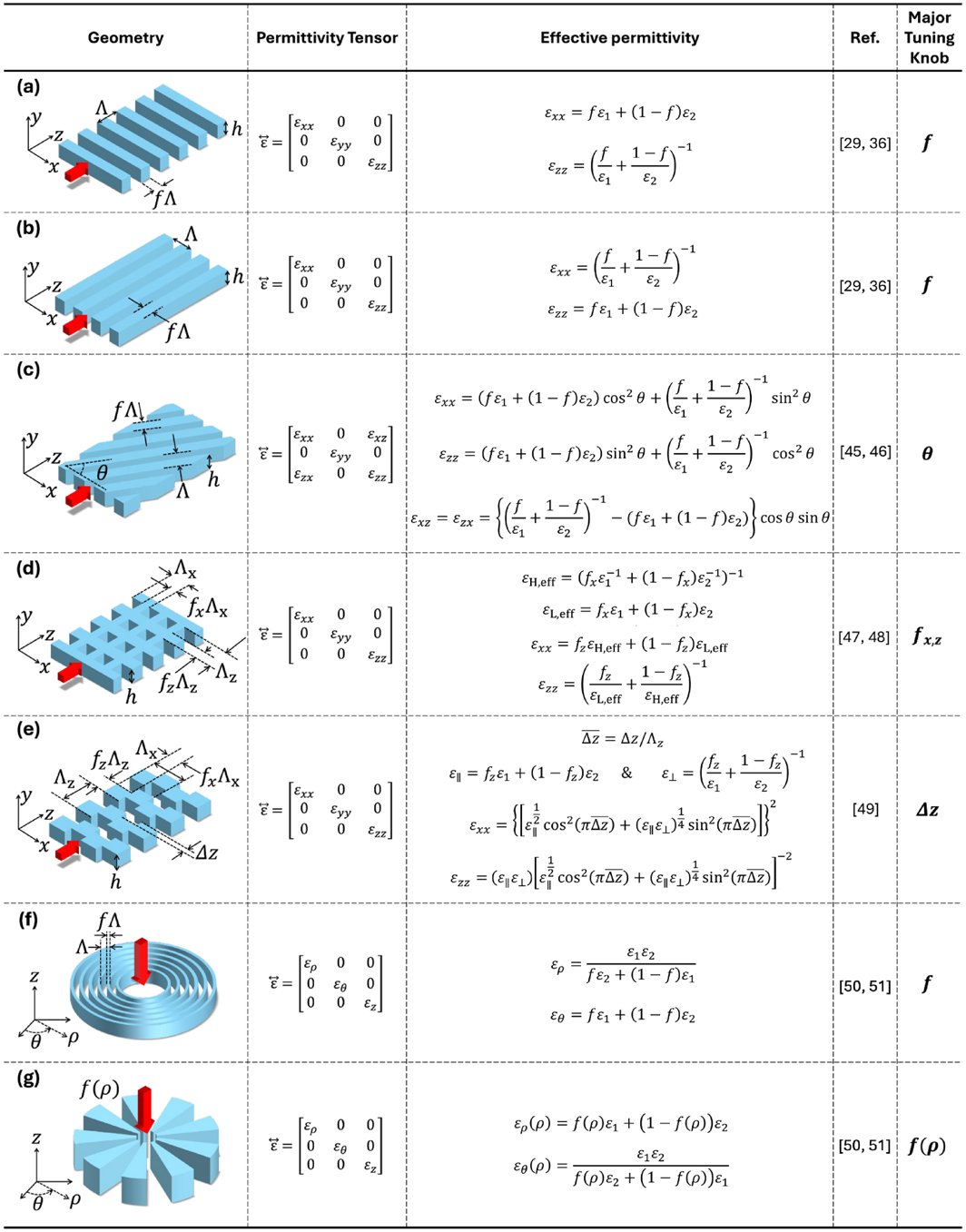# Correction to “Anisotropic Metamaterials for Scalable Photonic Integrated Circuits: A Review on Subwavelength Gratings for High‐Density Integration”

**DOI:** 10.1002/nap2.70128

**Published:** 2026-05-10

Y. Shin, et al., “Anisotropic Metamaterials for Scalable Photonic Integrated Circuits: A Review on Subwavelength Gratings for High‐Density Integration,” *Nanophotonics 14* (2025): 1311–1331, 10.1515/nanoph‐2024‐0627.

In the originally published version of Figure 1, there were typographical and labeling errors in the equations presented in panels (e), (f), and (g).

- In Figure 1e: The offset term in the cosine function was incorrectly written as Δz; this has been corrected to the normalized form $\bar{\Delta z}$ ($=\Delta z/\Lambda_{z}$) to accurately represent the effective permittivity. Additionally, the formula previously provided for permittivity (ϵ) was the expression for the refractive index (n); this has been replaced with the correct effective permittivity formula.
- In Figure 1f and g: The formulas for the radial ($\epsilon_{\rho }$) and azimuthal ($\epsilon_{\theta }$) permittivity components were inadvertently swapped. These equations have been transposed to align with the correct coordinate definitions.

The corrected Figure 1 is provided on the following page.

We apologize for this error.